# Eligibility for knee arthroplasty is associated with increased risk of acquired hallux valgus - a Mendelian randomized study

**DOI:** 10.1186/s12891-024-07458-2

**Published:** 2024-04-22

**Authors:** Zhijun Li, Zhengxuan Liu, Wei Shi, Xinyu Liang, Chunlei Xu, Kai Zhang, Hui Li, Huafeng Zhang

**Affiliations:** 1 Department of Orthopedics, Tianjin Medical University General Hospital, Tianjin, 300052 P. R. China; 2 Department of Orthopedics, Tianjin Hospital of ITCWM Nankai Hospital, Tianjin, 300052 P. R. China

**Keywords:** Acquired hallux valgus, Knee arthroplasty, Mendelian randomization, Osteoarthritis

## Abstract

**Objective:**

Clinically, it has been found that patients undergoing knee replacement have a high incidence of concomitant hallux valgus. In this study, we analyzed whether patients with osteoarthritis who underwent surgery and those patient who did not have surgery had an increased risk of hallux valgus by Mendelian randomization and performed reverse causal analysis.

**Design:**

Genomewide association study (GWAS) data for osteoarthritis, categorized by knee arthritis with joint replacement, knee arthritis without joint replacement, hip arthritis with joint replacement, and hip arthritis without joint replacement.And acquired hallux valgus were downloaded for Mendelian randomized studies. MR analysis was performed using inverse variance-weighted (IVW), weighted median, and MR-Egger methods. MR-egger regression, MR pleiotropic residuals and outliers (MR-presso), and Cochran's Q statistical methods were used to evaluate heterogeneity and pleiotropy.

**Results:**

The IVW results indicate that, compared to healthy individuals, patients who meet the criteria for knee osteoarthritis joint replacement surgery have a significantly higher risk of acquired hallux valgus. There were no significant causal relationships found for the remaining results. No significant heterogeneity or multiplicity was observed in all the Mr analyses.

**Conclusion:**

Our study supports the increased risk of acquired hallux valgus in patients eligible for knee replacement. There is necessary for clinicians to be concerned about the hallux valgus status of patients undergoing knee arthroplasty.

**Supplementary Information:**

The online version contains supplementary material available at 10.1186/s12891-024-07458-2.

## Introduction

Osteoarthritis (OA), one of the most prevalent degenerative joint diseases, affects various components of the joint, including the articular cartilage, subchondral bone, ligaments, joint capsule, synovial membrane, and surrounding muscles. This multifaceted condition involves structural changes that contribute to its progression [1]. OA can impact any joint in the body, but it commonly affects major weight-bearing joints such as the knee, as well as the hand, hip, and spine. This chronic condition leads to symptoms like joint pain, tenderness, swelling, and even deformity and these symptoms can significantly diminish a patient's quality of life and limit their mobility and functionality [2]. OA is the result of multiple risk factors, and age is the biggest risk factor for OA [3, 4]. The epidemiology of OA is intricate and influenced by various factors, including genetics, biology, and biomechanics. It is a prevalent and debilitating disease that poses a growing burden on patients, healthcare systems, and the overall social economy [1].

Hallux valgus (HV) is a frequently occurring deformity in the adult foot, with an incidence ranging from approximately 23% to 35%. This condition is characterized by the angulation, rotation, and lateral deviation of the great toe at the first metatarsophalangeal joint [5]. A recent review estimated that worldwide, the prevalence of HV is as high as 23% in people aged 18-65 years and 35% in people over 65 years [6]. The HV deformity can lead to functional disabilities, such as foot pain, compromised gait, diminished balance, and foot deformity,which can significantly impact a person's ability to walk comfortably and perform daily activities [7–10]. A study [11] revealed a noteworthy disparity in the occurrence of HV and knee osteoarthritis (KOA) among female patients. Furthermore, the study identified female gender and KOA as risk factors for HV. These findings suggest that being female and having KOA increase the likelihood of developing HV.It is also observed in clinical practice that there are similarities among individuals with KOA and HV. For instance, there is a higher prevalence of females, and factors such as age and lower limb deformities are commonly associated with both conditions [12–14].

Mendelian randomization (MR) is an analytical approach that applies Mendelian laws of inheritance to study the causal relationship between modifiable exposures and clinically relevant outcomes. This method utilizes single nucleotide polymorphisms (SNPs) as instrumental variables (IVs) to infer causation from observed associations [15]. Alleles are randomly separated during meiosis, so MR can reduce bias caused by confounders [16]. As a result, MR Is less susceptible to confounding factors and reverse causality than traditional observational methods. Standard MR Studies must satisfy three important assumptions: (1) Instrumental variables are strongly correlated with exposure factors; (2) Genetic variables were not associated with any confounders of exposure outcomes; (3) Instrumental variables can only affect outcomes through exposure [17].

In clinical practice, knee replacement patients have been observed to have a higher likelihood of developing hand osteoarthritis. This raises the question of whether osteoarthritis increases the risk of HV, or if HV itself increases the risk of knee osteoarthritis and hip osteoarthritis (HOA). To investigate this relationship, we conducted a Mendelian randomization (MR) analysis. Recent genome-wide association studies (GWAS) have identified distinct genetic polymorphisms associated with OA patients who have undergone joint replacement compared to those who have not [18]. Given that knee and hip OA are significant causes of pain and disability in older adults, no studies have compared foot characteristics in patients with HOA and KOA.

## Methods

### Data source

The OA data comes from a recent GWAS meta-analysis [18]. The data were divided into four subsets including OA site and whether joint replacement was performed: non-surgical KOA (38,626 case vs. 625,232 control), surgical KOA (22,525 case vs. 638,618 control), non-surgical HOA (17,847 case vs. 672,115 control), and surgical HOA (20,221 case vs. 626,610 control).The surgical group included patients who had undergone joint replacement surgery, while the non-surgical group consisted of patients who had not undergone joint replacement surgery. The control group consisted of healthy individuals. Different codes represent different diseases (ICD-10 M17.0 Primary osteoarthritis, bilateral; ICD-10 M17.1 Other primary arthrosis, etc.). Specific inclusion/exclusion criteria can be found in the supplementary material of the original article. GWAS data is available at https://www.decode.com/summarydata. The GWAS data for acquired HV are derived from the latest data from the fine database. The study included 12,055 patients with acquired HV and 202,617 controls. GWAS data can be downloaded from https://r7.finngen.fi/pheno/M13_HALLUXVALGUS. Criteria for diagnostic evaluation of the disease are given in the Finnish database.

### IV selection

**Fig. 1:**
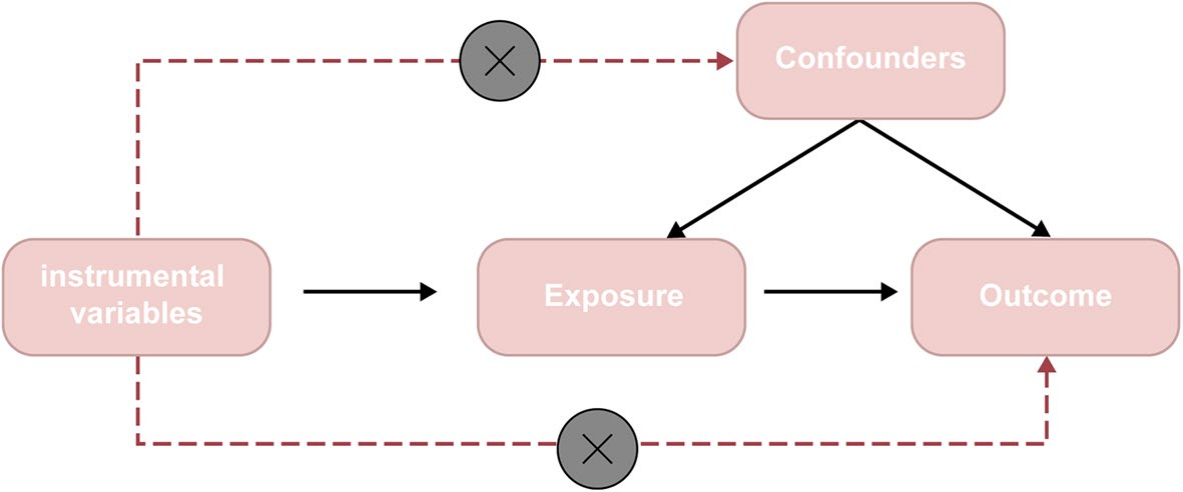
Selection of instrumental variables

The screening criteria for IV are: ① Based on the whole gene information of the European 1000 Genomes Project, the selected IVs had genome-wide significance (*P*<5×10-8); ② The physical distance between each two genes was >10 000 kb and the r2 threshold of LD between genes was <0.001 to exclude the effect of linkage disequilibrium; ③ We also removed the SNPs with palindrome allele; ④ The PhenoScanner database was used to further validate whether the above included SNPs loci were associated with other confounders. SNPs associated with BMI and weight were removed when OA was used as an outcome; ⑤ If the F-statistic of the SNPs is <10, it means that the SNPs have the possibility of weak instrumental variable bias, and then they will be excluded to avoid the impact on the results. The F-statistic of the single SNP: *F*=(β/SE)^2^ (β = effect size, SE = standard error) [19–21]. Please refer to Fig. 1 for the specific flowchart.

### Statistical analysis

In this study, the inverse variance weighted (IVW) method was mainly used [22]. If heterogeneity exists, a random effects model is selected [23]. Secondly, this study further complemented the above conclusions by using MR-Egger regression, Weighted median [24–26]. Only when the results (OR values) of the above three methods are consistent, the results are stable.

### Horizontal pleiotropy and heterogeneity tests

In this study, outliers were detected using the MR-PRESSO method.If there are outliers, they are eliminated and the analysis is repeated."Leave-one-out" sensitivity analyses were performed by removing a single SNP at a time to assess whether the variant drove the association between the exposure and outcome variables.Secondly, in order to determine whether the MR Analysis had horizontal pleiotropy, the MR-Egger intercept detection was also carried out in this study. If the intercept term in the MR-Egger intercept analysis has obvious statistical significance (*P*<0.05), it indicates that the study has obvious horizontal pleiotropy. Finally, this study also usd Cochran's Q statistic to detect heterogeneity. If Cochran's Q statistic test is statistically significant (*P*<0.05), it proves that the analysis results are heterogeneous.

The correlations with a *P*-value < 0.05 were considered to be statistically significant. R version 4.2.0 was used for all statistical analyses. R packages such as Twosample MR and MR-PRESSO were used.

## Results

### Effect of OA on HV

When performing joint replacement with HOA as the exposure, we found that rs3814333 was associated with high heel phenotype and excluded it. The remaining instrumental variables had no significant direct or indirect correlation with HV. All eligible instrumental variables can be found in the GWAS data for HV. We selected 15, 4, 29, and 2 SNPS as instrumental variables in the GWAS data of surgical KOA, non-surgical KOA, surgical HOA, and non-surgical HOA, respectively. Details on tool variables are provided in the supplemental documents.

**Fig. 2:**
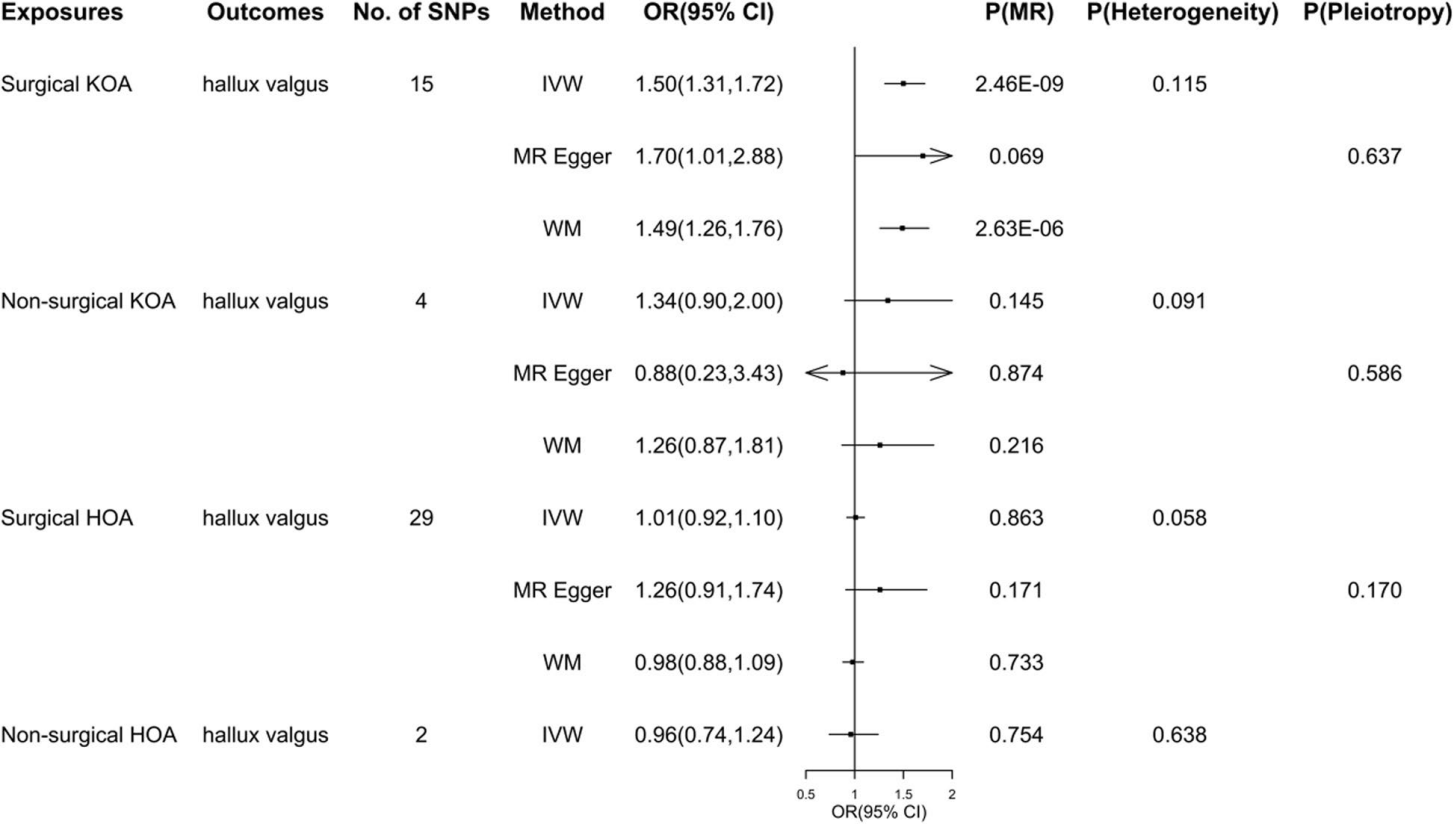
Results of MR analysis of the effect of OA on acquired HV, as well as heterogeneity and pleiotropy analyses

Since MR-egger and WM methods require at least 3 SNPS, only IVW method was used for analysis in the group non-surgical HOA.The results showed that the incidence of HV in group surgical KOA increased significantly. IVW results of four MR Analyses are as follows: surgical KOA - acquired HV (OR: 1.50; 95%CI (1.31-1.72); *P*<0.001), non-surgical KOA - acquired HV(OR: 1.34; 95%CI (0.90-2.00); *P*=0.145), surgical HOA - acquired HV (OR: 1.01; 95%CI (0.92-1.10); *P*=0.863), non-surgical HOA - acquired HV(OR: 0.96; 95%CI (0.74-1.24); *P*=0.754). There was no significant heterogeneity or pleiotropy in all the results (Fig. 2). The results of leave-one-out analysis are shown in Fig. 4.

### Effect of HV on OA

When HV deformity was used as exposure, rs22433 was associated with body weight and rs1317349 with BMI,which was removed. All eligible SNPS can be found in the OA data.

**Fig. 3:**
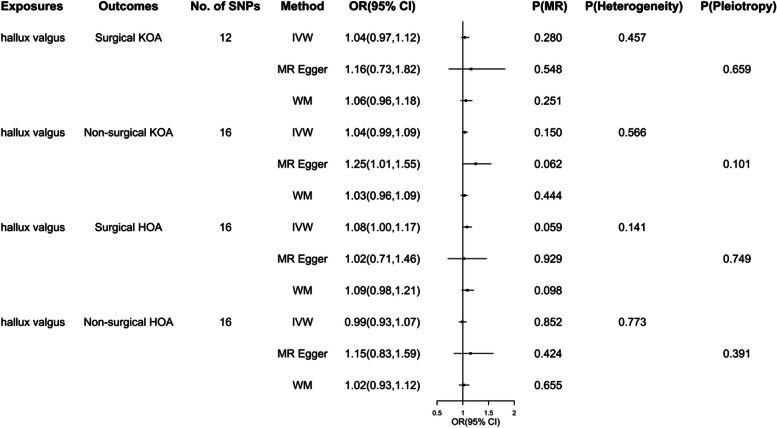
Results of MR analysis of the effect of acquired HV on OA, as well as heterogeneity and pleiotropy analyses

**Fig. 4:**
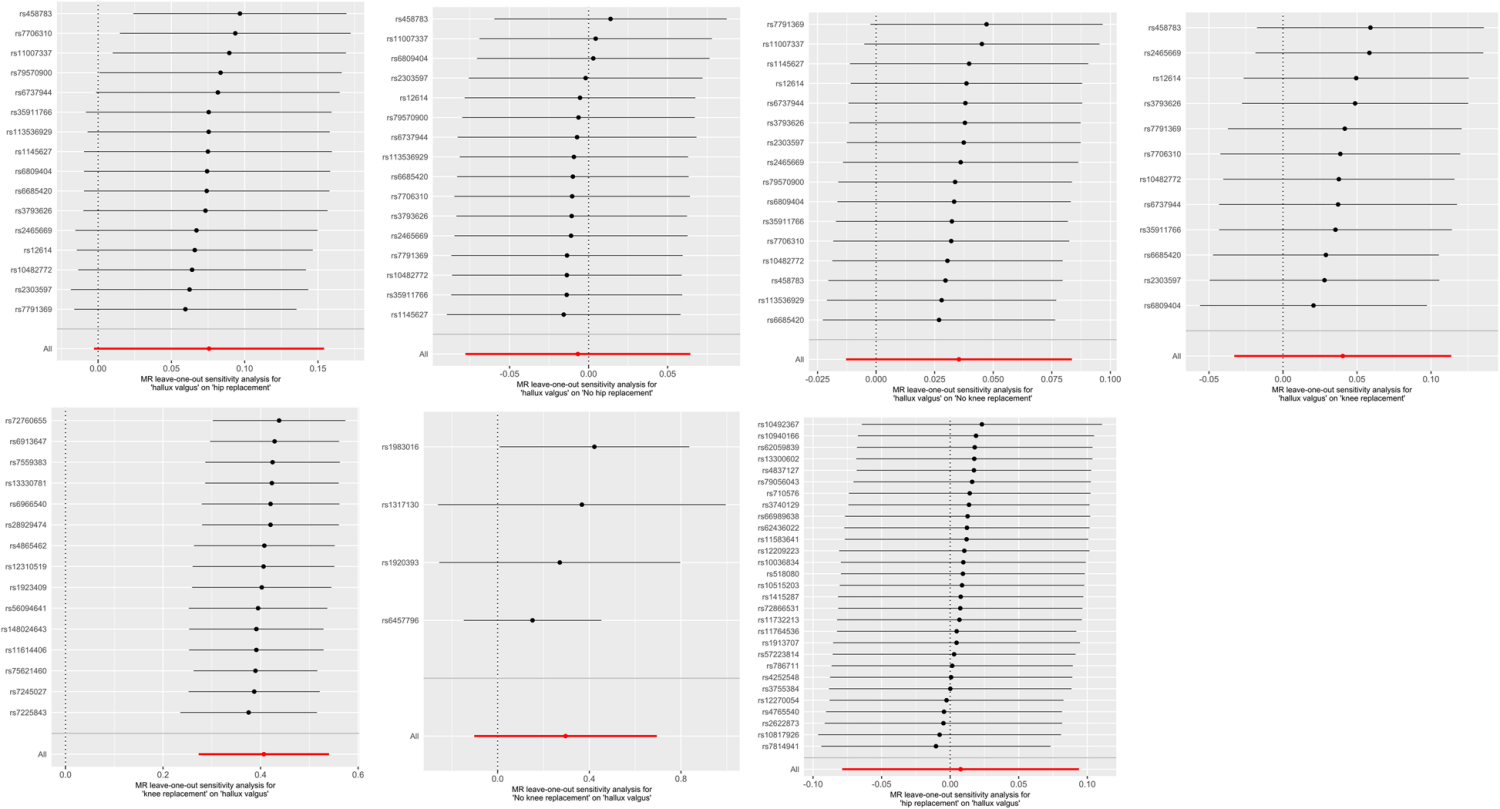
Results of leave-one-out analysis for each group

The results showed that acquired HV can not increase the risk of OA. IVW results of four MR Analyses are as follows: acquired HV - surgical KOA(OR:1.04; 95%CI(0.96-1.12); *P*=0.280), acquired HV - non-surgical KOA(OR:1.04; 95%CI(0.99-1.09); *P*=0.150), acquired HV - surgical HOA(OR:1.08; 95%CI(1.00-1.17); *P*=0.059), acquired HV - non-surgical HOA(OR: 0.99; 95%CI(0.93-1.07); *P*=0.852). There was also no significant heterogeneity or pleiotropy in all the results which suggested that the MR findings were robust (Fig. 3). The results of leave-one-out analysis are shown in Fig. 4.

## Discussion

To our knowledge, this study represents the first MR analysis utilizing genetic data from a publicly available database to investigate the association between osteoarthritis (OA) patients (knee and hip) who underwent joint replacement and those who did not, with respect to the occurrence of acquired hand osteoarthritis (HV). The bidirectional MR analysis revealed a significant increase in the risk of HV among the knee replacement group. However, there was no observed increase in HV risk among patients who did not undergo knee replacement or those with hip osteoarthritis (HOA). Additionally, the occurrence of acquired HV did not increase the risk of developing knee or hip osteoarthritis, whether surgery was performed or not. It is important to note that the division of data into joint replacement and non-joint replacement groups does not imply a causal relationship between joint replacement surgery and the occurrence of acquired HV. Rather, it suggests that individuals eligible for knee replacement are at a higher risk of acquiring HV compared to the general population. Furthermore, our findings do not indicate that severe OA poses a greater risk for acquired HV than mild OA. Our grouping merely highlights that patients meeting the criteria for knee replacement have an increased risk of acquiring HV compared to individuals without such indications.

The risk factors of OA can be divided into individual factors and biomechanical factors, and the interaction between the two is complex. Individual factors include age, gender, obesity, genetics, diet, etc. Biomechanical factors include joint shape, limb alignment, abnormal joint load (such as unequal length of lower limbs), muscle function and physical activity [27]. A study [28] conducted across the United States identified age as the most influential risk factor for osteoarthritis, with the prevalence of OA increasing as individuals grow older. This finding suggests a possible decline in the regenerative capacity of cartilage and the accumulation of various risk factors associated with aging.Hammertoe deformityis influenced by a variety of risk factors, including genetic susceptibility, structural factors, gender, age, body mass index (BMI), foot pain, flat feet, and shoe shape [29]. These factors can contribute to the development and progression of HV.The frequent wearing of high heels has been associated with an increased prevalence of HV. To ensure the accuracy of our analysis, we excluded the genetic variant rs3814333, which is specifically associated with the high heel phenotype. However, it is important to note that the relationship between HV and osteoarthritis is not clearly established as a causal one. To investigate this relationship, we used a robust analytical method called Mendelian randomization to minimize bias caused by confounding factors.

In our study, we observed a significant association between HV and OA patients who had undergone knee replacement surgery. This finding suggests a potential link between the two conditions. However, further clinical studies are necessary to confirm this conclusion. While our analysis provides valuable insights, additional research is needed to fully understand the relationship between HV and OA.

During clinical practice, it is often observed that patients with knee osteoarthritis may exhibit varus or valgus deformities of the knee joint, along with the possibility of flexion contracture. Additionally, it is noticeable that patients with KOA have a higher likelihood of experiencing abnormal force distribution compared to those with healthy knee joints. This observation suggests that these factors may contribute to an increased risk of developing acquired hallux valgus. In a cross-sectional study conducted by Golightly et al., it was discovered that hallux valgus may be indicative of adult bone development or an early sign of osteoarthritis in the first metatarsophalangeal joint [30–33].

Based on our findings, clinicians should pay more attention to the status of HV. when performing joint replacement surgery on patients with KOA and decide whether to intervene. Patients with HV have varying degrees of foot deformities, which can also cause pain and poor foot appearance [7]. Our findings serve as a reminder to clinicians to identify and intervene at an earlier stage in patients with osteoarthritis and hallux valgus. Additionally, it is crucial to recognize that the joints in each segment of the lower limb function independently and have an impact on one another. Therefore, when performing a specific joint operation, it is essential to take into account the overall alteration in the lower limb's force distribution in order to determine the most suitable surgical approach.

This study has obvious advantages over observational studies.By means of Mendelian randomization, we make full use of genetic information as a tool to study causality, and the results are statistically strong.Clinicians can avoid conducting complex and expensive randomized clinical trials and no longer need to worry about common clinical ethical issues [34]. Nevertheless, there are certain limitations to consider in this study. Firstly, the majority of participants in this study were of European descent, which may limit the generalizability of the findings to other ethnic groups. While this characteristic might help reduce bias resulting from population stratification, it does not establish the applicability of the results to diverse populations.Secondly, as in all Mendelian randomization (MR) studies, this research is unable to address unobserved confounding variables, potentially introducing bias into the results.Furthermore, since the reasons why non-surgical OA patients did not undergo surgery were not specified during the inclusion of the population in the genome-wide association study (GWAS), the differences between non-surgical OA patients and surgical OA patients cannot be directly explained. It is important to note that non-surgical OA does not necessarily equate to mild OA, as patients with non-surgical OA may have severe contraindications for surgery. Therefore, our results do not provide evidence that the severity of OA is directly associated with an increased risk of HV.And due to the influence of the GWAS inclusion sample, there may be non-surgical patients who meet the indications for surgery but are not treated surgically, and this type of error is again unavoidable. We used GWAS data of acquired HV, not congenital HV. Whether congenital HV increases the risk of OA was not analyzed.

## Conclusion

Overall, our study supports an increased risk of HV in patients eligible for knee replacement. For patients scheduled for knee arthroplasty, it is necessary for clinicians to pay attention to their HV status, fully consider the alignment of the patient's lower limbs, and improve preoperative preparation. For patients with HV, clinicians can examine the knee joint of patients at the same time, according to the imaging examination and clinical symptoms of patients, early intervention for patients with imaging abnormalities without clinical symptoms, correct unhealthy lifestyles, reduce risk factors, so as to slow down the progression of knee OA.

### Supplementary Information

**Supplementary material 1.**

## Data Availability

There is no use of raw, unprocessed data in this study. The datasets mentioned in this study can be found in online repositories. The OA GWAS data source: https://www.decode.com/summarydata. The acquired hallux valgus GWAS data source: https://r7.finngen.fi/pheno/M13_HALLUXVALGUS.

## Abbreviations

GWAS: Genomewide association study
IVW: Inverse variance-weighted
OA: Osteoarthritis
HV: Hallux valgus
KOA: Knee osteoarthritis
MR: Mendelian randomization
SNPs: Single nucleotide polymorphisms
IV: Instrumental variables
HOA: Hip osteoarthritis
